# Robotic traction-assisted en bloc endoscopic resection of a large laterally spreading tumor in the descending duodenum

**DOI:** 10.1055/a-2885-8089

**Published:** 2026-07-08

**Authors:** Haoran Liu, Lihe Liu, Hanchao Pan, Lijuan Qian, Zhaoyang Zou, Rui Li, Airong Wu

**Affiliations:** 1 Department of Gastroenterology 74566 The First Affiliated Hospital of Soochow UniversitySuzhouChina

Endoscopic submucosal dissection (ESD) in the descending duodenum is technically challenging due to the thin wall, rich vascular supply, and complex anatomy, leading to a higher risk of adverse events compared to other gastrointestinal sites. EndoFaster (ROBO Med, Shenzhen, China), a flexible auxiliary single-arm transluminal endoscopic robot, has been validated for safety and feasibility in assisting endoscopic resection in difficult locations such as the gastric fundus and the lower rectum. 1​ 2​ ​3​ We report the application of EndoFaster-assisted ESD for a laterally spreading tumor (LST) in the distal descending duodenum.

**Fig. 1:**
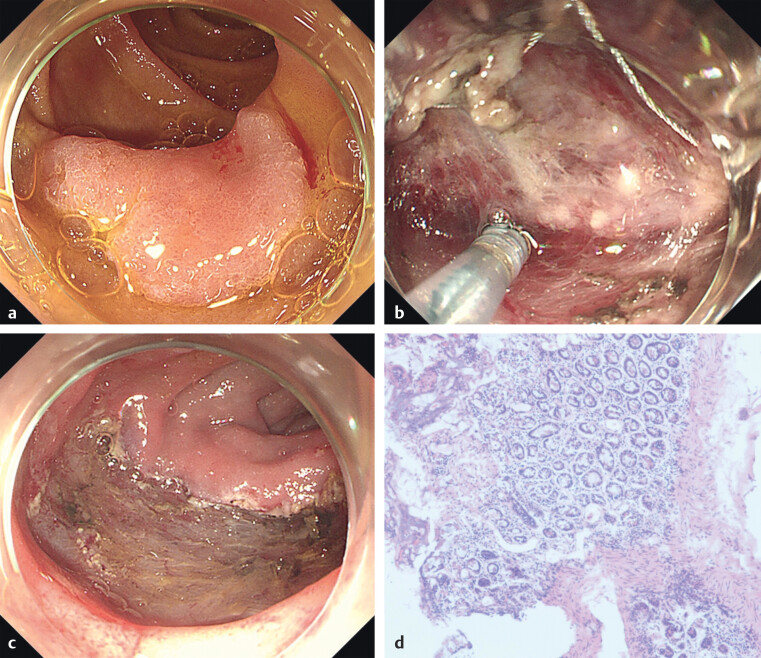
Endoscopic appearance and histopathological findings. ( **a** ) Gastroscopy revealed a LST (approximately 3.0×3.5 cm) in the descending duodenum. ( **b** ) The application of a robotic traction system provided continuous upward traction and improved visualization of the submucosal layer during ESD. ( **c** ) Post-resection ulcer bed after en bloc removal, with no evidence of perforation or active bleeding. ( **d** ) Histopathological examination (HE,×100) revealed a tubular adenoma with focal low-grade intraepithelial neoplasia; no tumor involvement was observed at the lateral or vertical resection margins, supporting a R0 resection.

A 50-year-old man underwent gastroscopy, revealing a 3.0×3.5 cm LST involving one-third of the circumference in the descending duodenum near the horizontal portion ( Fig. 1a ). Biopsy confirmed tubular adenoma with low-grade intraepithelial neoplasia. ESD was successfully performed with the assistance of EndoFaster for traction. After indigo carmine spraying, submucosal injection and circumferential incision, the grasper was mounted at the 12 o’clock position of the endoscope tip. The forceps grasped the oral mucosal edge, providing continuous upward traction perpendicular to the muscularis propria, which significantly enhanced submucosal visualization ( Fig. 1b ). This approach facilitated efficient dissection and allowed for prompt hemostasis. The total dissection time was 8.6 minutes, with no perforation or muscle layer injury ( Fig. 1c; Video 1 ). The patient had an uneventful postoperative course and was discharged on the 5th day after the procedure. Histopathology confirmed R0 resection ( Fig. 1d ).

**Video 1** En bloc endoscopic resection of a large laterally spreading tumor in the descending duodenum using a robotic traction system.


A multicenter retrospective study in China evaluated the clinical outcomes of conventional ESD for duodenal LSTs>15 mm in diameter. The results showed a median procedure time of 45.5 minutes (range 20–117 minutes) for 51 lesions. 4​ These findings indicate that robot-assisted traction, by clearly exposing the submucosal field, improves both the efficiency and the safety of endoscopic resection for duodenal LSTs, thereby providing strong support for the future development of small-bowel ESD techniques.

Endoscopy_UCTN_Code_TTT_1AO_2AG_3AD
